# 

**Published:** 1969-07

**Authors:** 


					Vi V
July 1969
The
Bristol
Medico-Chin
Journal
NATIONAL LIBRARY OF MEDICINE
-?'l--:  1 recd. AUG 8 1969
__Tn~;rs?:
??OL-'.-r.; -~5\\
INDEX VQ i
INPUT
- ?>nrru
. . - _ lii
: ;.1.2C
RU?H:
incorporating the medical journal of the south west
Vol. 84 (Hi) No 311
INDEX MED1CUS

				

